# Supplementary material for: MetaRibo-Seq measures translation in microbiomes
Source: Nat Commun. 2020 Jun 29;11:3268. doi: 10.1038/s41467-020-17081-z (PMC7324362; doi:10.1038/s41467-020-17081-z)
Supplement: Supplementary file 10 — Supplementary Data 7 [file 41467_2020_17081_MOESM10_ESM.zip › File2/Confidence_High_Taxonomy/135964_out.krona.html]

Javascript must be enabled to view this page.

members
magnitude
magnitudeUnassigned
count
unassigned
taxon
rank

135964\_out

10


SRS016989\_contig\_number\_6534SRS021948\_contig\_number\_contig-100\_7802.45485SRS077641\_contig\_number\_contig-100\_2387.98903SRS143148\_contig\_number\_contig-100\_28115.28116
4

2
6
superkingdom

5
phylum
1239

class
3
186801

186802
3
order

1
family
186803

1
genus
841

166486

SRS105153\_contig\_number\_37567
1
species

1
family
31979

genus
1
1485

1262777

SRS098073\_contig\_number\_contig-100\_5029.5030
1
species

186806
1
family

genus
1
1730

142586
1

SRS148784\_contig\_number\_8212
species

2
class
909932

1843488
order
2

909930
family
2

genus
2
33024

species

SRS015431\_contig\_number\_42544SRS019068\_contig\_number\_110857
2
626940

1
phylum
976

768503
1
class

768507
order
1

SRS149181\_contig\_number\_contig-100\_2826.122932
